# Network-timing-dependent plasticity

**DOI:** 10.3389/fncel.2015.00220

**Published:** 2015-06-09

**Authors:** Vincent Delattre, Daniel Keller, Matthew Perich, Henry Markram, Eilif B. Muller

**Affiliations:** ^1^Laboratory of Neural Microcircuitry, Brain and Mind Institute, École Polytechnique Fédérale de LausanneLausanne, Switzerland; ^2^Center for Brain Simulation, École Polytechnique Fédérale de LausanneGeneva, Switzerland; ^3^Department of Biomedical Engineering, Northwestern University, EvanstonIL, USA

**Keywords:** synaptic plasticity, patch-clamp, acute brain slices, somatosensory cortex, STDP, self-organized criticality, neural networks simulations

## Abstract

Bursts of activity in networks of neurons are thought to convey salient information and drive synaptic plasticity. Here we report that network bursts also exert a profound effect on Spike-Timing-Dependent Plasticity (STDP). In acute slices of juvenile rat somatosensory cortex we paired a network burst, which alone induced long-term depression (LTD), with STDP-induced long-term potentiation (LTP) and LTD. We observed that STDP-induced LTP was either unaffected, blocked or flipped into LTD by the network burst, and that STDP-induced LTD was either saturated or flipped into LTP, depending on the relative timing of the network burst with respect to spike coincidences of the STDP event. We hypothesized that network bursts flip STDP-induced LTP to LTD by depleting resources needed for LTP and therefore developed a resource-dependent STDP learning rule. In a model neural network under the influence of the proposed resource-dependent STDP rule, we found that excitatory synaptic coupling was homeostatically regulated to produce power law distributed burst amplitudes reflecting self-organized criticality, a state that ensures optimal information coding.

## Introduction

Periods of synchronous neuronal firing, or bursts of action potentials (APs) in populations of neurons, are ubiquitous in the central nervous system. Bursts can induce long-lasting changes in synaptic efficacy depending on the frequency of bursting, with long-term depression (LTD) being induced by low frequency bursts and long-term potentiation (LTP) being induced at higher frequencies (Bliss and Lomo, 1973; Lynch et al., 1983; Stanton and Sejnowski, 1989; Dudek and Bear, 1992) with notable exceptions (Coesmans et al., 2004). This phenomenon led to the well-known Bienenstock Cooper and Munro (BCM) model of synaptic plasticity (Bienenstock et al., 1982). The relative timing of single spikes generated in connected pairs of neurons can also induce LTP and LTD (Markram et al., 1997; Bi and Poo, 1998), which has led to the well-known Spike-Timing-Dependent Plasticity (STDP) model of synaptic plasticity (Markram et al., 2011). The manner in which these two induction protocols for synaptic plasticity interact is unclear, and it remains to be seen if they can be unified under a common mechanism.

According to the so-called calcium hypothesis, synaptic changes are thought to be determined by the magnitude and time-course of the transient influx of calcium into the synaptic spine induced by pre- and post-synaptic spiking (Bear et al., 1987; Shouval and Kalantzis, 2005; Nevian and Sakmann, 2006; Graupner and Brunel, 2010, 2012). Large calcium influxes are thought to induce potentiation, whereas moderate and prolonged calcium influxes are thought to induce depression (Bienenstock et al., 1982; Nevian and Sakmann, 2006). A network burst induced transient reduction in extracellular calcium would reduce the magnitude of calcium influx into the spine approximately proportionally (Egelman and Montague, 1999; Wiest et al., 2000), and could subsequently alter the outcome of plasticity. In particular, spiking motifs yielding LTP could instead yield LTD when embedded in a network burst.

This shifting of the direction of plasticity in bursting networks toward LTD is an interesting observation, as it is a possible mechanism for counteracting run-away potentiation in networks of neurons with on-going synaptic plasticity. Increases in synaptic coupling between excitatory neurons are known to induce a transition to bursting activity regimes, as has been reported in previous theoretical studies (Tsodyks et al., 2000; Kudela et al., 2003) and under pathological experimental conditions where synaptic up-scaling was induced by activity deprivation (Trasande and Ramirez, 2007). While homeostatic mechanisms have been proposed to down-regulate synaptic strengths if neuronal firing rates become excessive (Turrigiano et al., 1998; Trasande and Ramirez, 2007), such mechanisms have been shown to be insufficient to maintain network stability in simulations of networks of neurons incorporating empirically constrained STDP models at excitatory synapses (Zenke et al., 2013). One important reason for this is that such mechanisms are insensitive to the transition to the network bursting state, which occurs with only minor changes in neuronal firing rates. The proposed interplay between network bursting activity and STDP could provide negative feedback allowing fine homeostatic control to be maintained in the presence of on-going synaptic plasticity, and thus to maintain states of criticality observed in cortical networks (Beggs and Plenz, 2003; Priesemann et al., 2009, 2013).

To gain insight into the proposed interaction of STDP and network bursting activity, we investigated *in vitro* the effect of precisely timed network bursts on STDP at excitatory synaptic inputs to layer 5 pyramidal neurons where the STDP phenomenon was first reported (Markram et al., 1997). STDP protocols known to induce LTP and LTD were applied, and network bursts were induced at precise timings before, during, or after the STDP pairing protocols using the electrodes of a multi-electrode array (MEA) located in layer 5. The pairing of STDP events with network bursts can influence the plasticity outcome by altering the timing relationship in the pre–post spike motif due to the additional spikes, and by changes in context due to the network burst (such as voltage, competition for resources, etc.). To separate the former effects from the latter, we performed burst-spike-substitution (BSS) experiments whereby the MEA burst was replaced with an excitatory postsynaptic potential (EPSP) paired with a simultaneous post-synaptic AP.

Our main experimental finding is that certain specific timings of network bursts relative to the STDP events can induce flips of LTD into LTP and LTP into LTD, which cannot be accounted for by the BSS protocols, and thus on pre–post spiking alone. We propose that the observed flips are manifestations of positive and negative synaptic cooperativity, respectively, for which a number of mechanisms have been proposed. We hypothesize that *negative cooperativity* could be due to the depletion of critical resources needed for LTP, perhaps through the depletion of an intracellular messenger (Fonseca et al., 2004), or the transient reduction of extracellular calcium at synaptic junctions immediately following network bursting (Egelman and Montague, 1999; Wiest et al., 2000).

We further hypothesize that the observed negative cooperativity could have an important role in the maintenance of the excitation–inhibition balance and of network criticality in the presence of on-going synaptic plasticity. To evaluate this hypothesis, we employ simulations of networks of neurons incorporating an empirically constrained STDP rule (Morrison et al., 2007), and augment it with a resource depletion term implementing a shift of STDP outcomes from LTP to LTD when embedded in a network burst. Networks including the resource depletion term are found to induce a transition to a state of criticality in the network (Beggs and Plenz, 2003; Priesemann et al., 2009, 2013). The proposed resource-dependent interaction between network activity and STDP therefore represents a novel mechanism for the homeostatic regulation of the network activity regime.

## Materials and Methods

### Electrophysiology

In accordance with the Swiss national and institutional guidelines, 300 μm thick sagittal brain slices were prepared from somatosensory cortex of postnatal days 13–17 Wistar rats of either sex in iced artificial cerebrospinal fluid (ACSF) containing (in mM) 125 NaCl, 2.5 KCl, 25 D-glucose, 25 NaHCO_3_, 1.25 NaH_2_PO_4_, 2 CaCl_2_, and 1 MgCl_2_; all chemicals from Sigma–Aldrich (St. Louis, MO, USA or Merck, Darmstadt, Germany), using a HR2 vibratome (Sigmann Elektronik, Heidelberg, Germany). The primary somatosensory cortex was manually dissected and isolated to obtain rectangular slices of 5–7 mm width and containing the neocortex in its entire height. Optimal slices, with apical cell dendrites running parallel to the slice surface, were selected for recordings. Slices were incubated at 22°C for 30–60 min until mounting in the recording chamber. Slices were mounted on a 3D-MEA with 60 pyramidal platinum electrodes (electrode basis: 40 μm × 40 μm, electrode height: 50–70 μm, electrode interspacing: 200 μm; Qwane Bioscience SA, Lausanne, Switzerland) after evaporation of a mounting solution of 0.14 mg/L nitrocellulose in an ethanol (99%) – methanol (1%) mixture. Cells were visualized by infrared differential interference contrast video microscopy using a camera (VX 55, Till Photonics, Gräfelfing, Germany) mounted on an upright microscope (BX 51WI, Olympus, FI, Japan) fitted with a 40× objective (LUMPLAN, Olympus). Whole-cell recordings were performed using Axopatch 200B amplifiers (Molecular Devices, Union City, CA, USA). Data acquisition, sampled at 5–10 kHz, was performed via an ITC-18 board (Instrutech Co, Port Washington, NY, USA), connected to a computer running IgorPro (Wavemetrics, Portland, OR, USA). The voltage signal was filtered with a 2 kHz Bessel filter. Multiple somatic whole cell recordings (1–3 cells simultaneously) were performed using patch pipettes pulled with a P-97 Flaming/Brown micropipette puller (Sutter Instruments Co, Novato, CA, USA) with an initial resistance of 8–10 M Ω. Patch pipettes were filled with standard intracellular solution (ICS) containing (in mM): 110 K-gluconate, 10 KCl, 4 ATP-Mg, 10 phosphocreatine, 0.3 GTP, 10 *N*-2-hydroxyethylpiperazine-*N*′-2-ethanesulfonic acid (pH 7.3), and 0.5% biocytin. Recordings were not corrected for the liquid junction potential between ACSF and ICS (–14 mV). Variation in the cell input resistance was measured from beginning to end of the experiment, and all cells having a change in input resistance greater than 33% were excluded. Cell access resistance was typically less than 20 MΩ.

### Electrical Stimulation

**FIGURE 1:**
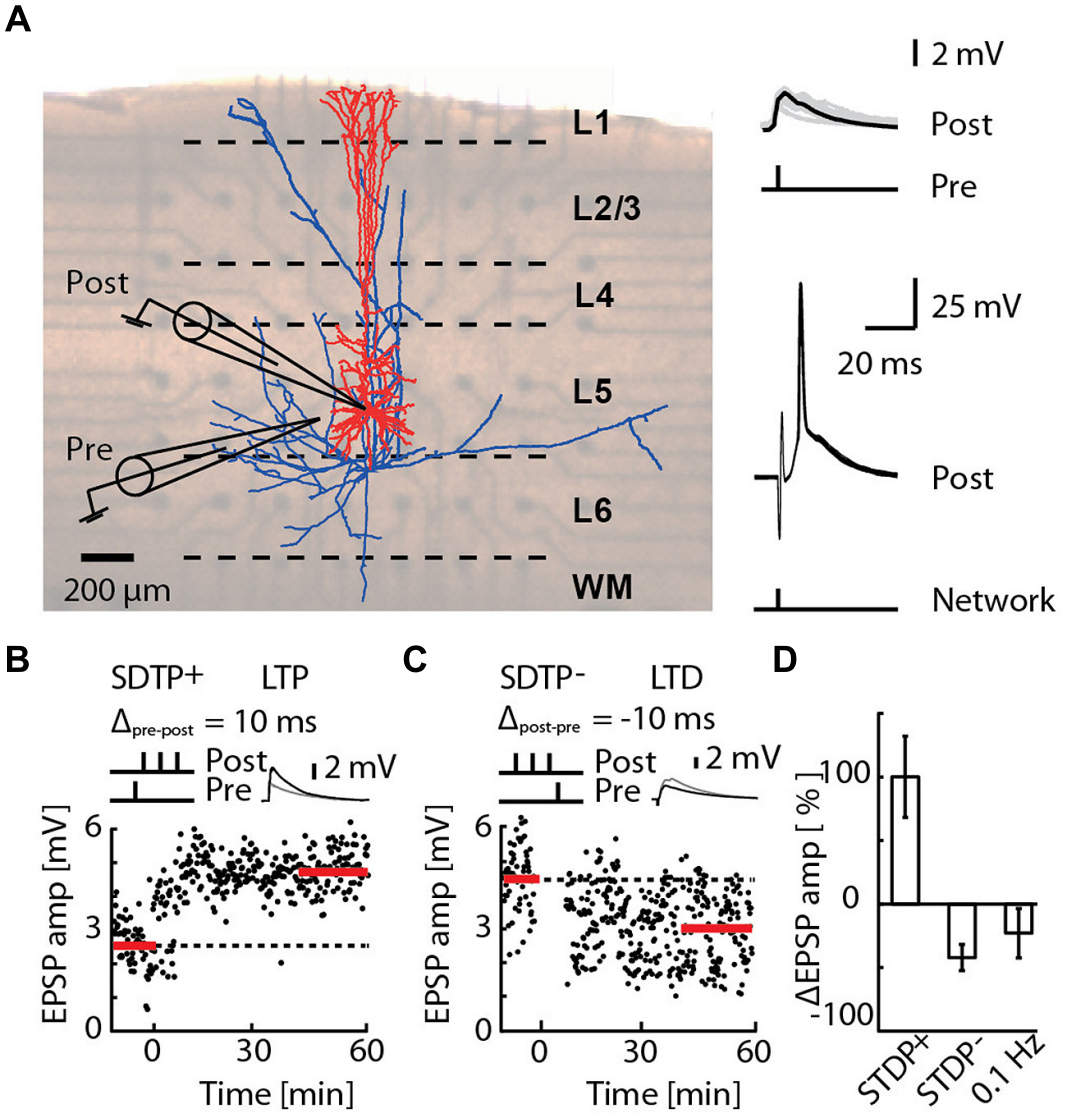
**Induction of long-term potentiation (LTP) and long-term depression (LTD) by pairing an EPSP with a short burst of action potentials (APs), or by network bursting. (A)** Cortical slice mounted on a 3D-multi-electrode array (MEA), with a reconstruction of a layer 5 pyramidal neuron (blue axons, red dendrites) overlaid (left). A whole-cell patch of the pyramidal neuron (post) receiving an EPSP (pre) evoked by extracellular electrical stimulation (upper-right). The post-synaptic responses due to network bursts evoked by MEA stimulation in the region of layer 5 are overlaid for 30 repetitions (lower-right). **(B)** A typical recording for the STDP^+^ paradigm (STDP^+^; black circles). EPSP amplitude was measured every 10 s (baseline and final amplitude indicated by the red line). **(C)** A typical recording for the STDP^-^ paradigm (STDP^-^; black circles). **(D)** Mean change in EPSP amplitude for STDP^+^ (ΔEPSP amp. = 103 ± 33%, *n* = 11), STDP^-^ (ΔEPSP amp. = -44 ± 10%, *n* = 6) and 0.1 Hz network bursting (ΔEPSP amp. = -25 ± 20%; *n* = 9).

A STDP protocol known to induce LTP (the STDP^+^ event) was applied as a 50 Hz train of three APs with a single evoked EPSP 10 ms earlier (Nevian and Sakmann, 2006; **Figure 1B**). A STDP protocol known to induce LTD (the STDP^-^ event) was applied as a 50 Hz train of three APs with a single evoked EPSP 10 ms later (Nevian and Sakmann, 2006; **Figure 1C**). EPSPs were evoked by extracellular stimulation using an extracellular pipette located near the basal dendrites of the patched-cell, and paired with APs evoked with supra-threshold intracellular current injection, as previously described (Nevian and Sakmann, 2006). Pairings were repeated 60 times at a frequency of 0.1 Hz. EPSPs were monitored at a frequency of 0.1 Hz, for 10 min prior to pairing to record the baseline and for more than 1 h post pairing. Network bursts were evoked by extra-cellular electrical stimulation of layer 5 (STG2008 stimulator, Multi Channel System, Reutlingen, Germany) using a 3D-MEA (electrode basis: 40 μm × 40 μm, electrode height: 50–70 μm, inter-electrode spacing: 200 μm; Qwane Bioscience SA, Lausanne, Switzerland). Stimulation strength was tuned to trigger a single AP in every patched cell (1–2 V biphasic pulses; 1 ms duration in each polarity). On average, the network burst failed to evoke a spike in 6.8% of the cases, triggered a single spike in 89.2% of the cases, and two spikes in 4% of the cases (Supplementary Figure S1). We never observed a network burst causing more than two spikes in the patched cells. The latency of the first evoked spike was 3.7 ± 0.2 ms (range 1.4–10.8 ms, *n* = 3960 network bursts recorded in 66 cells). Due to the symmetry of the MEA evoked network burst, pre-synaptic spiking in excitatory cells during the burst is assumed to mirror post-synaptic spiking. The time interval Δ*T* between EPSP and the network burst was defined as the time between EPSP digital trigger and the network burst digital trigger.

Bursts were evoked before (–20 ms), simultaneous to (0 ms) or at the end of (50 ms) an STDP^+^ event, and at the beginning of (–50 ms), simultaneous to (0 ms), or after (20 ms) an STDP^-^ event. The timing of the burst with respect to the STDP^-^ protocol to was chosen to exactly mirror all tested protocols for LTP. The combined burst-STDP event pairing was applied at a frequency of 0.1 Hz.

Burst-spike-substitution experiments replaced the network burst with an EPSP paired with a simultaneous post-synaptic AP for burst-STDP pairings, to mirror the pre- and post-synaptic spiking as seen by a synapse during a burst-STDP event (assuming the predominant case above that bursts trigger a single pre- and post-synaptic spike), but without the network context.

### Experimental Data Analysis and Statistics

Experimental data analysis was performed in Matlab (The MathWorks, Inc., Natick, MA, USA) with custom scripts. EPSP amplitude was monitored for an hour and 20 min. Baseline EPSP was acquired over the first 10 min, followed by 10 min of pairing, as described above. The final EPSP amplitude was averaged over the last 20 min of recordings. EPSP failure or EPSPs that caused the cell to spike were excluded from the analysis. However, if after the pairing, a cell fired 100% of the time following the EPSP onset within an averaging time window (20 repetitions), we assumed a strong potentiation to have occurred and set the synaptic gain to a value of 5 for this time period. Data are presented as the mean ± SEM. Paired Student’s *t*-tests were applied as statistical tests, and statistical significance was asserted for: ^∗^*p* < 0.05; ^∗∗^*p* < 0.01; ^∗∗∗^*p* < 0.001.

### Network Simulations

We simulated a network of 1000 integrate-and-fire (IF) neurons (of which 80% are excitatory and 20% inhibitory) arranged on a 10 × 10 × 10 lattice, corresponding to a 200 µm × 200 µm × 200 µm volume of cortex in an active state. Neuron parameters were fit to publicly available Hodgkin–Huxley type neuron models (Destexhe et al., 1998) for f-vs-I curves and noise current injections. Excitatory neurons contained a spike triggered conductance inducing spike-frequency adaptation (Muller et al., 2007). Each neuron had 1000 excitatory (AMPA) and 250 inhibitory (GABA_A_) conductance-based synapses with a peak conductance of 2 nS (except plastic synapses) and time constants of 1.5 ms and 10 ms, respectively. Consistent with anatomy, 10–20% of the synaptic inputs originated from neurons inside the network (140 exc. → exc., 200 exc. → inh., 50 inh. → exc., 50 inh. → inh.), and delays were computed as *dβ* (1+*ξ*), where *d* is the distance, β = 0.25 ms/unit lattice and *ξ* is a random number drawn from an exponential distribution with mean of 0.2. Extrinsic input was modeled by Poisson processes with firing rates parameterized separately for excitatory and inhibitory input, 6 and 10.5 Hz, respectively. These rates were determined numerically to be consistent with excitatory and inhibitory model neuron firing rates resulting from application of exclusively Poissonian input at these rates at all synapses. Neuronal properties in the network are consistent with the “high-conductance state” (Destexhe et al., 2003). We used the Power-law STDP rule (Morrison et al., 2007) parameterized for cortical (Froemke and Dan, 2002) conductance-based synapses to achieve a mean of ∼1.9 nS under extrinsic input alone as follows: *τ*_+_ = 14 ms; *τ_-_* = 34 ms; *w*_0_ = 4.29 × 10^-2^ nS; µ = 0.4; λ = 0.1; and α = 4.8 × 10^-2^. For the computation of STDP time differences, connection transmission delays were treated as half axonal and half dendritic. Where stated, the STDP rule was augmented with a model for activity-dependent resource availability as described in the main text. The network and neuron models were implemented using the PyNN modeling language (Davison et al., 2009) with the NEURON simulator backend (Hines and Carnevale, 1997) and are publicly available at: https://neuralensemble.org/svn/PyNN/trunk/examples/iaf_sfa_relref/

### Resource Dependent STDP

To implement the hypothesized effects of resource depletion, such as extracellular calcium, on STDP as a mechanism to flip LTP into LTD, we added a resource depletion term to a standard STDP learning rule (Morrison et al., 2007) in the network model. We modeled resource depletion caused by network activity by assuming the equilibrated resource availability for any fixed average network firing rate, *α,* has the form η_0_(α) = (1+α/*k*)^-1^, where *k* is the depletion rate constant (*k* was assigned to 20 Hz to allow for a 50% resource depletion during sustained 20 Hz network activity). The dynamic resource availability η*(t)* was then computed by low pass-filtering η_0_(α(*t*)) as follows:

$$\frac{d}{dt}\eta \left(t\right)=\frac{\eta_{0}\left(\alpha \left(t\right)\right)-\eta \left(t\right)}{\eta_{0}\left(\alpha \left(t\right)\right)\cdot \tau_{\eta }},$$

where α*(t)* is a continuous estimator of the average networking firing rate (low-pass filtered network spiking with a filter time constant *τ*_α_ = 2.5 ms and normalized by network size), *τ_η_* is the recovery time constant of the resource availability (assumed to be 100 ms) in the absence of network activity, and the factor of η_0_(α(*t*)) in the denominator ensures that depletion is fast while recovery is slow. Biologically, the hypothesized resource depletion is likely to be a local phenomenon, but the extent of the locality remains unknown. As further experiments reveal the actual distance over which the network could act on a synapse, sub-volumes can be defined and η*(t)* computed for each voxel in the context of the whole network. We, however, did not define sub-volumes of the network and considered the firing of all neurons when computing η*(t),* representing the average resource availability for the entire network volume.

To regulate LTP induced by STDP^+^ events, we scaled the computed synaptic weight change Δ*W*_+_ by the resource availability η*(t)* and implemented the scaling as

$$\Delta W_{+}^{\prime }=\gamma \left(\eta \left(t\right)\right)\cdot \Delta W_{+},$$

where γ*(η)* is a sigmoidal resource modulation function,

$$\gamma \left(\eta \left(t\right)\right)=\frac{2}{1+\exp\left(\frac{\eta^{*}-\eta \left(t\right)}{m}\right)}-1,$$

with η^∗^ = 0.6 defining the LTP–LTD reversal point and *m = 0.03* the steepness of the reversal. In this model, synapses active at the onset of bursts consume resources needed to potentiate, thus forcing later activated synapses to depress.

### Criticality Analysis

We fit a power-law using methods described in Clauset et al. (2009) to the cumulative burst size distribution of the network PSTH (dt = 2 ms) normalized by the SD of the activity of the sub-threshold network model (ω = 1.2 nS) without plasticity. The threshold for burst detection was set to the mean network firing rate normalized by the SD of the network firing rate when ω = 1.2 nS plus two. The branching parameter (σ) was computed as previously described (Beggs and Plenz, 2003; Priesemann et al., 2009).

## Results

### Network-Timing-Dependent Plasticity

We investigated in acute slices of juvenile rats mounted on a 3D-MEA (**Figure 1A**) the effect of precisely timed network bursts on STDP at excitatory synaptic inputs to layer 5 pyramidal neurons. STDP protocols known to induce LTP (the STDP^+^ event) and LTD (the STDP^-^ event) were applied as a 50 Hz train of three APs with, respectively, a single evoked EPSP 10 ms earlier or later (Nevian and Sakmann, 2006; **Figure 1**, see Materials and Methods). The STDP^+^ event reliably induced LTP (**Figures 1B,D**; ΔEPSP = 103 ± 33%, *n* = 11) and the STDP^-^ event reliably induced LTD (**Figures 1C,D**; ΔEPSP = –44 ± 10%, *n* = 6), also as previously reported (Nevian and Sakmann, 2006). Network bursts were evoked by extra-cellular electrical stimulation of layer 5 using a 3D-MEA (see Materials and Methods). On average, the network burst failed to evoke a spike in 6.8% of cases, triggered a single spike in 89.2% of cases, and two spikes in 4% of cases (Supplementary Figure S1). We never observed a network burst causing more than two spikes in the patched cells. The latency of the first evoked spike was 3.7 ± 0.2 ms (range 1.4–10.8 ms, *n* = 3960 network bursts recorded in 66 cells). Bursts alone evoked at 0.1 Hz induced LTD in seven out of nine cells (ΔEPSP = –41 ± 12%, *p* < 4e-3, one sample *t*-test to baseline 0%), whereas one cell exhibited no significant change (ΔEPSP = 19 ± 20%), and one cell expressed LTP (ΔEPSP = 111 ± 15%). Pooling all these cells in a single group led to a relatively high variability, and a skewed distribution (**Figure 1D**; ΔEPSP = –25 ± 20%; *n* = 9). Taken together, these results indicate that bursts alone evoked at 0.1 Hz generally induced LTD, consistent with previous reports for 1 Hz evocation of population EPSPs in hippocampal slices (Stanton and Sejnowski, 1989; Dudek and Bear, 1992), however, a small sub-population of cells expressing LTP cannot be excluded.

**FIGURE 2:**
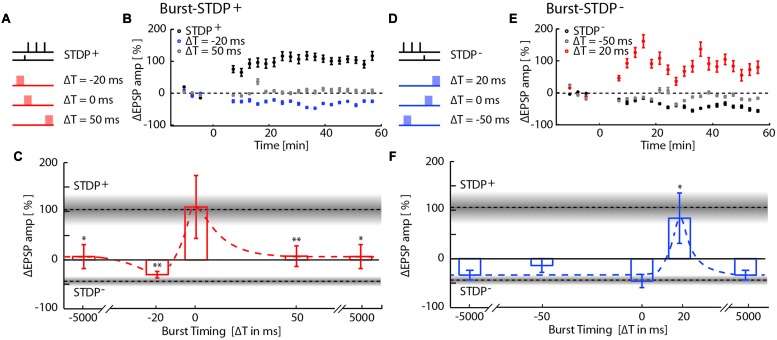
**Network-timing-dependent modulation of Spike-Timing-Dependent Plasticity (STDP). (A)** Pairing of STDP^+^ with MEA evoked network bursts at various relative timings with respect to the presynaptic STDP^+^ input. **(B)** EPSP amplitude changes due to burst-STDP^+^ pairings when the burst precedes (Δ*T* = -20 ms; blue circles) or follows (Δ*T* = 50 ms; gray circles) the STDP^+^ event (black circles). **(C)** Summary of changes in EPSP amplitude for the various STDP^+^ protocols. Dotted lines and gray shaded areas show the mean ± SEM EPSP amplitude change induced by STDP^+^ and STDP^-^. Depending on its relative timing, the burst either flipped LTP to LTD (burst preceding; Δ*T* = -20 ms), blocked LTP (burst following; Δ*T* = 50 ms, ± 5 s), or had no effect on the STDP pairing (simultaneous burst; Δ*T* = 0 ms). **(D)** Pairing of STDP^-^ with network bursts at various timings. **(E)** EPSP amplitude changes due to burst-STDP^-^ pairings when the burst precedes (Δ*T* = -50 ms; gray circles) or follows (Δ*T* = 20 ms; red circles) the STDP^-^ event (black circles). **(F)** Summary of changes in EPSP amplitude for the various STDP^-^ protocols. Dotted lines and gray shaded areas show the mean ± SEM EPSP amplitude change induced by STDP^+^ and STDP^-^. STDP^-^ induced LTD is unaffected unless the burst shortly follows the STDP^-^ event (Δ*T* = 20 ms).

We then examined the interaction between burst-induced LTD and STDP^+^ events (**Figures 2A–C**). The LTP induced by STDP^+^ remained unchanged when bursts coincided with the STDP^+^ events (Δ*T* = 0; ΔEPSP = 109 ± 64%, *p* = 0.17; *n* = 8), indicating that plasticity driven by relative spike timing in a specific synaptic pathway is unaffected by simultaneous network bursts. This result also indicates that LTD normally induced by such network bursts is blocked by simultaneous STDP^+^ events. However, when bursts preceded the STDP^+^ event by 20 ms, LTP flipped into LTD with a magnitude comparable to that of the burst-induced LTD (Δ*T* = –20 ms; ΔEPSP = –21 ± 7%, *p* = 3e^-4^, *n* = 7), indicating that immediately preceding network bursts block STDP^+^-induced LTP. Conversely, STDP^+^ events fail to block LTD induced by preceding bursts. Burst- and STDP^+^-induced plasticity mutually canceled when bursts were evoked 5s before or after STDP^+^ events (Δ*T* = ±5000 ms; ΔEPSP = 7 ± 24%, *p* = 2.8e^-2^; *n* = 8) or 50 ms after the STDP^+^ event (Δ*T* = +50 ms; ΔEPSP = 8 ± 21%, *p* = 9.3e^-3^; *n* = 14). All statistical tests comparing each protocol to baseline and to other protocols are provided in Supplementary Table S1.

We next tested for interactions between burst-induced LTD and STDP^-^ events (**Figures 2D–F**). Bursts that were simultaneous, 50 ms before and 5s before or after STDP^-^ events had no cumulative effect on the LTD induced by the STDP^-^ events, indicating that LTD is saturated. This saturation suggests that burst- and STDP^-^-induced LTD share expression mechanisms. Surprisingly, LTD flipped into LTP when bursts immediately followed STDP^-^ events (Δ*T* = 20 ms, 81 ± 50%, *p* = 3.5e^-2^, *n* = 9), indicating that the combination of LTD expression mechanisms induced by both burst and STDP^-^ events results in the expression of LTP.

**FIGURE 3:**
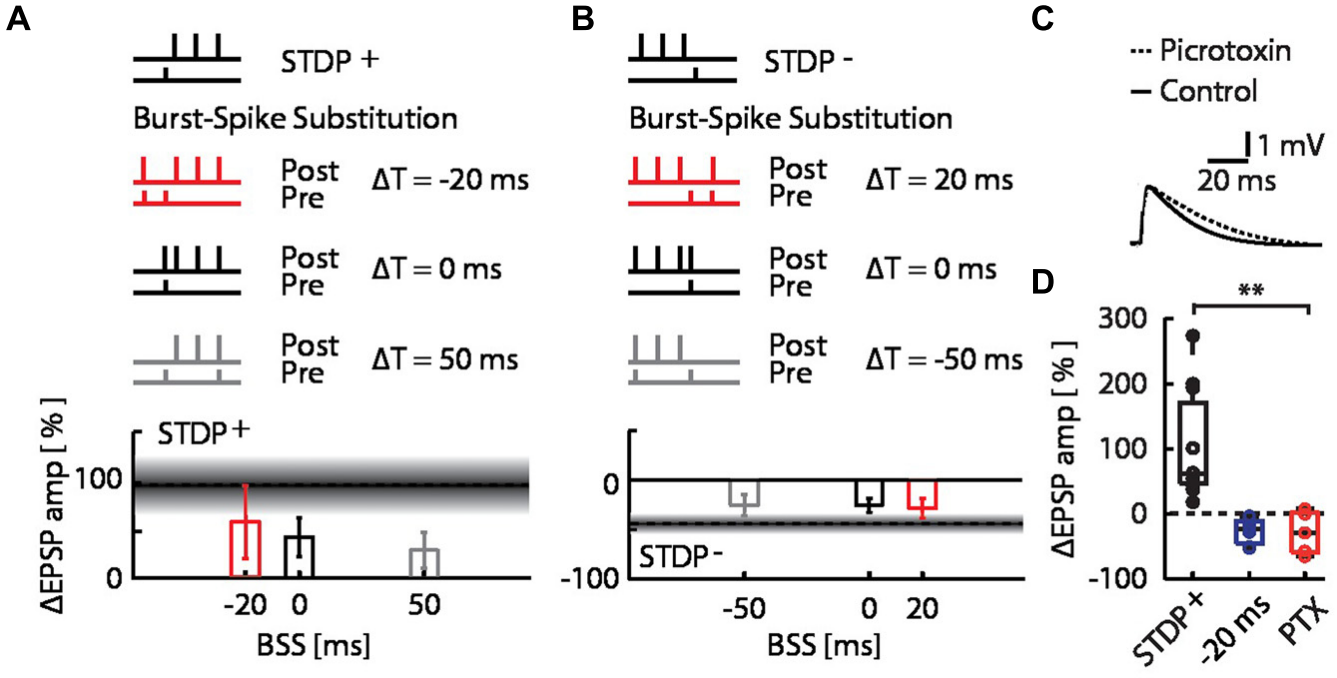
**Burst-spike-substitution (BSS) protocols do not explain the flip from LTP to LTD and LTD to LTP, and inhibitory circuits do not contribute to the flip from LTP to LTD. (A)** EPSP amplitude changes for BSS protocols of burst-STDP^+^ pairings, with simultaneous AP and EPSP at Δ*T* = -20 ms (red), Δ*T* = 0 ms (black) and Δ*T* = 50 ms (gray). All timings yielded LTP. **(B)** EPSP amplitude changes for BSS protocols of burst-STDP^-^ pairings, with simultaneous AP and EPSP at Δ*T* = 20 ms (red), Δ*T* = 0 ms (black) and Δ*T* = -50 ms (gray). All timings yielded LTD. **(C)** Average normalized EPSP baseline waveforms for control cells, and cells with intracellular picrotoxin (PTX) reveal the effect of PTX on the evoked response by the stimulation with the extracellular pipette. **(D)** EPSP amplitude change for the STDP^+^ event (black) with the network burst at Δ*T* = -20 ms (blue), and with the network burst at Δ*T* = -20 ms with PTX (red).

The pairing of STDP events with network bursts can influence the plasticity outcome by alterations of the timing relationship in the pre–post spike motif due to the additional spikes, and by changes in context due to the network burst. To determine whether the observed interaction between bursts and STDP events can be explained entirely by the single pre-synaptic and single post-synaptic spikes added to the STDP pairing protocol by the MEA stimulation, we repeated the burst-STDP pairing experiments substituting the burst with a single EPSP simultaneous to an AP (**Figures 3A,B**; BSS, see Materials and Methods). In terms of pre- and post-synaptic spiking, this BSS is equivalent to MEA stimulation (see Materials and Methods and Supplementary Figure S1). BSS could not account for the flip of LTP into LTD due to a burst 20 ms before the STDP^+^ event (**Figure 3A**; red bar), nor the flip of LTD into LTP due to a burst 20 ms after the STDP^-^ event (**Figure 3B**, red bar). All other BSS timings yielded changes in EPSP amplitudes that were consistent with their respective burst-STDP pairings (**Figures 3A,B**). These data imply that multiple inputs to the neuron from the bursting network are required to induce the observed flips in directionality of plasticity.

Finally, we examined whether inhibitory synaptic inputs activated by the burst played a role in the observed flip of LTP into LTD. We repeated the burst-STDP^+^ experiment with the burst 20 ms before the STDP^+^ event, while blocking inhibitory currents in the patched cells with intracellular picrotoxin (PTX; **Figure 3C**; Paille et al., 2013). We found that the bursts still flipped the LTP into LTD (**Figure 3D**; burst at Δ*T* = –20 ms + PTX; open red circles; ΔEPSP amp = –26 ± 14%, *p* = 0.18 against STDP^+^ and *p* = 0.38 against burst + STDP^+^, *n* = 5), indicating that inhibitory inputs do not play a significant role in burst-dependent STDP.

Taken together, these data suggest that the observed flips of LTD into LTP and LTP into LTD could be manifestations of positive and negative synaptic cooperativity, respectively. We hypothesized that the flip from LTP into LTD (negative cooperativity) could be due to the depletion of critical resources needed for LTP.

### Resource-Dependent Regulation of STDP

**FIGURE 4:**
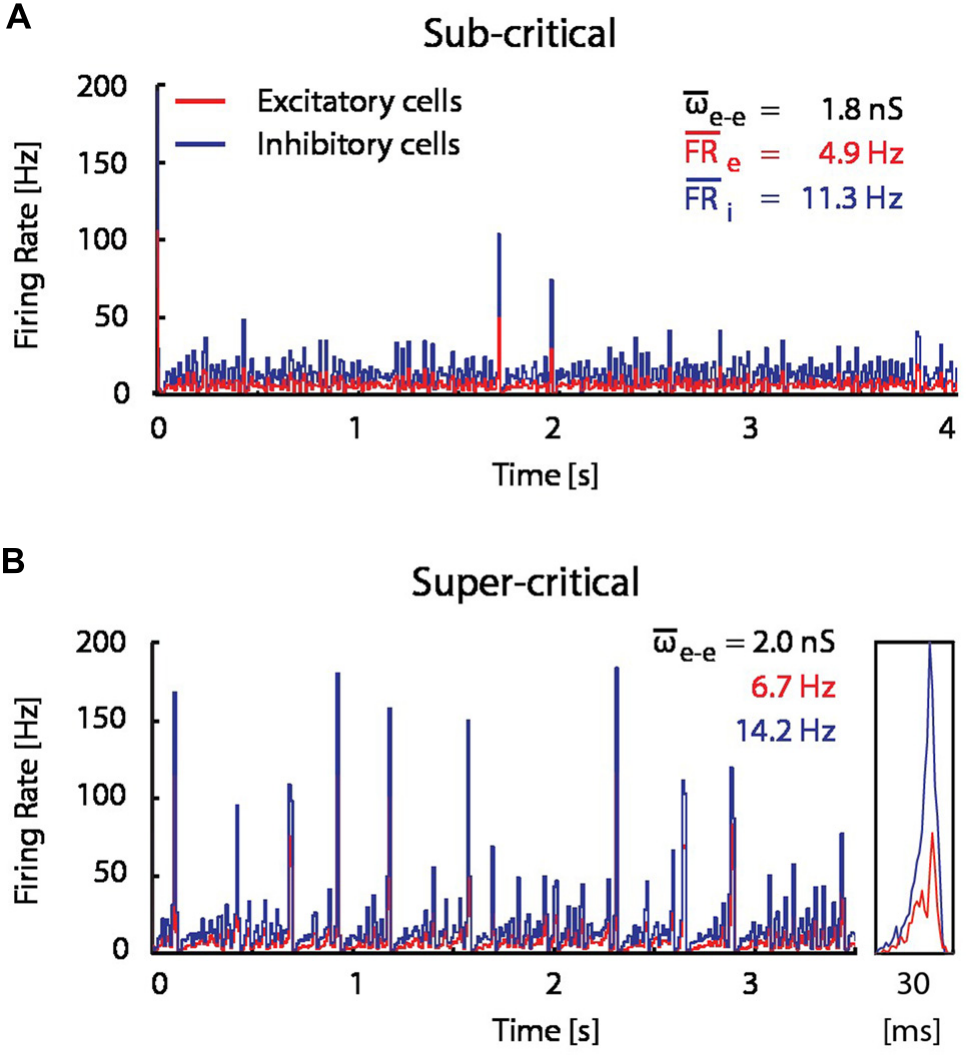
**A modest increase in excitatory coupling leads to spontaneous network bursting.** Firing rate of recurrent randomly connected network of 1000 integrate-and-fire (IF) neurons with 20% inhibitory cells in an active state (see Materials and Methods); 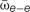 is the mean excitatory coupling, 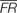
_e_ and 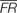
_i_ are the mean firing rate of excitatory (red) and inhibitory (blue) cells in the network, respectively. The simulated network changes its state from sub-critical **(A)**, to super-critical (spontaneous periodic bursting, **B**) after a 10% increase of mean synaptic weight for excitatory–excitatory connections. (Inset) Example of a typical network burst is shown to the right.

In order to assess the significance of the observed flip from LTP into LTD, and the hypothesized resource depletion on STDP, we proposed a resource-dependent STDP learning rule (see Materials and Methods), and examined its implications in network simulations. We simulated the network dynamics and evolution of synaptic weight distributions without STDP, with STDP and with resource-dependent STDP in a simplified network model consisting of 1000 IF neurons (80% excitatory, 20% inhibitory; see Materials and Methods). Without STDP, network dynamics are highly sensitive to the mean excitatory–excitatory synaptic coupling. For example, a mere 10% increase in coupling is sufficient to drive the network from a sub-critical regime exhibiting aperiodic occurrence of spontaneous bursts at low frequencies (**Figure 4A**), to a supra-critical regime exhibiting frequent spontaneous and periodic network bursts (**Figure 4B**; Beggs and Plenz, 2003; Shew and Plenz, 2013). This transition from sub- to supra-critical activity regimes has been reported in previous theoretical studies (Tsodyks et al., 2000; Kudela et al., 2003) and under pathological experimental conditions where synaptic up-scaling was induced by activity deprivation (Trasande and Ramirez, 2007).

**FIGURE 5:**
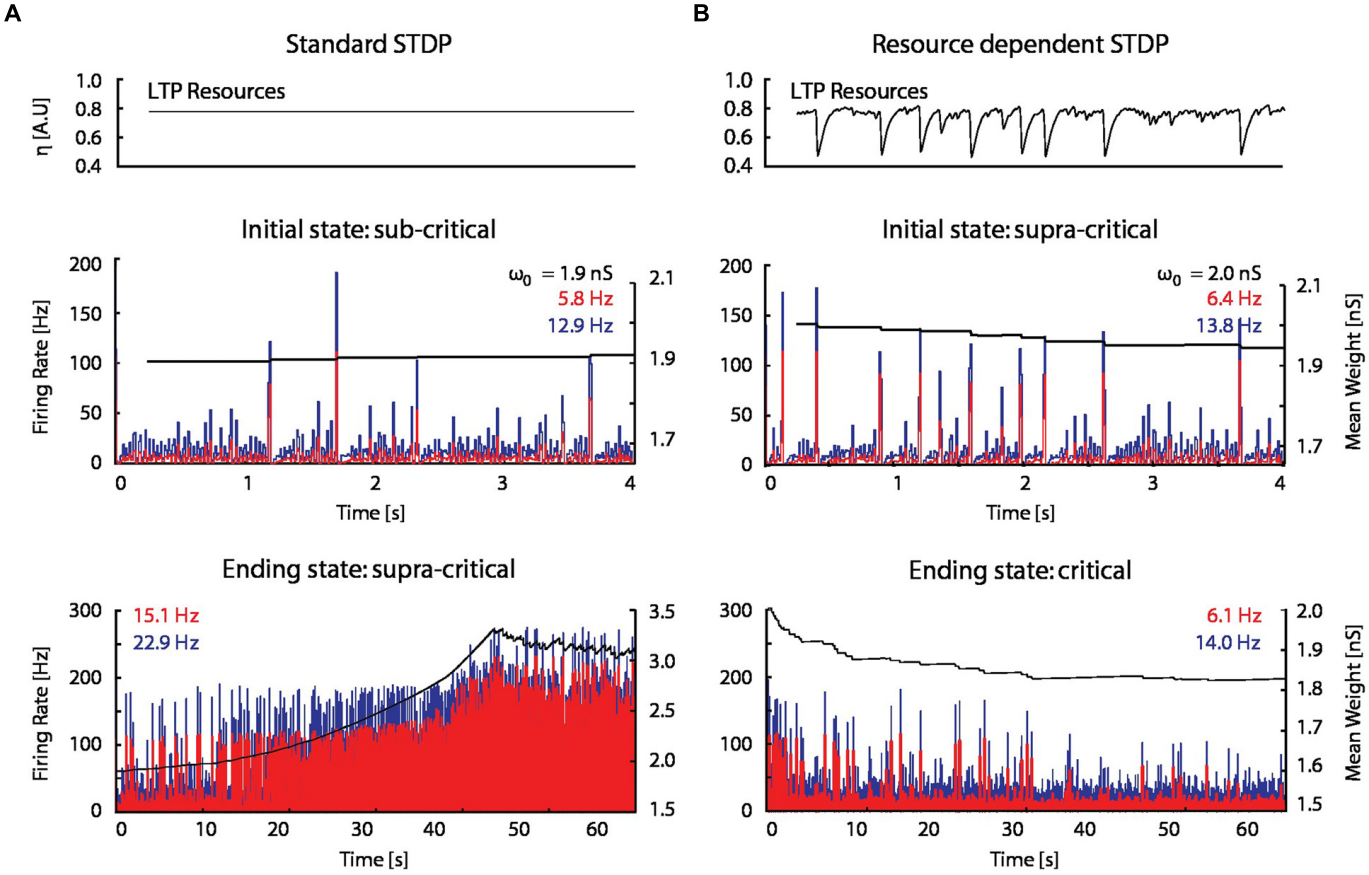
**A STDP model with activity-dependent resource consumption counter-balances runaway potentiation. (A)** With a standard STDP rule, the firing rate and mean synaptic weight of a network initialized in a sub-critical state drift toward non-physiological values in a supra-critical state; ω_0_ is the initial mean excitatory–excitatory synaptic strength. **(B)** The resource-dependent STDP rule drives networks initialized in a super-critical state toward a critical state.

When STDP was introduced into the model network (see Materials and Methods), spontaneous network bursts resulted in more LTP than LTD on average, which gradually increased excitatory–excitatory coupling, and in turn led to an increase in burst frequency and amplitude. This positive feedback drove synaptic weights and network activity to non-physiological regimes (**Figure 5A**).

**FIGURE 6:**
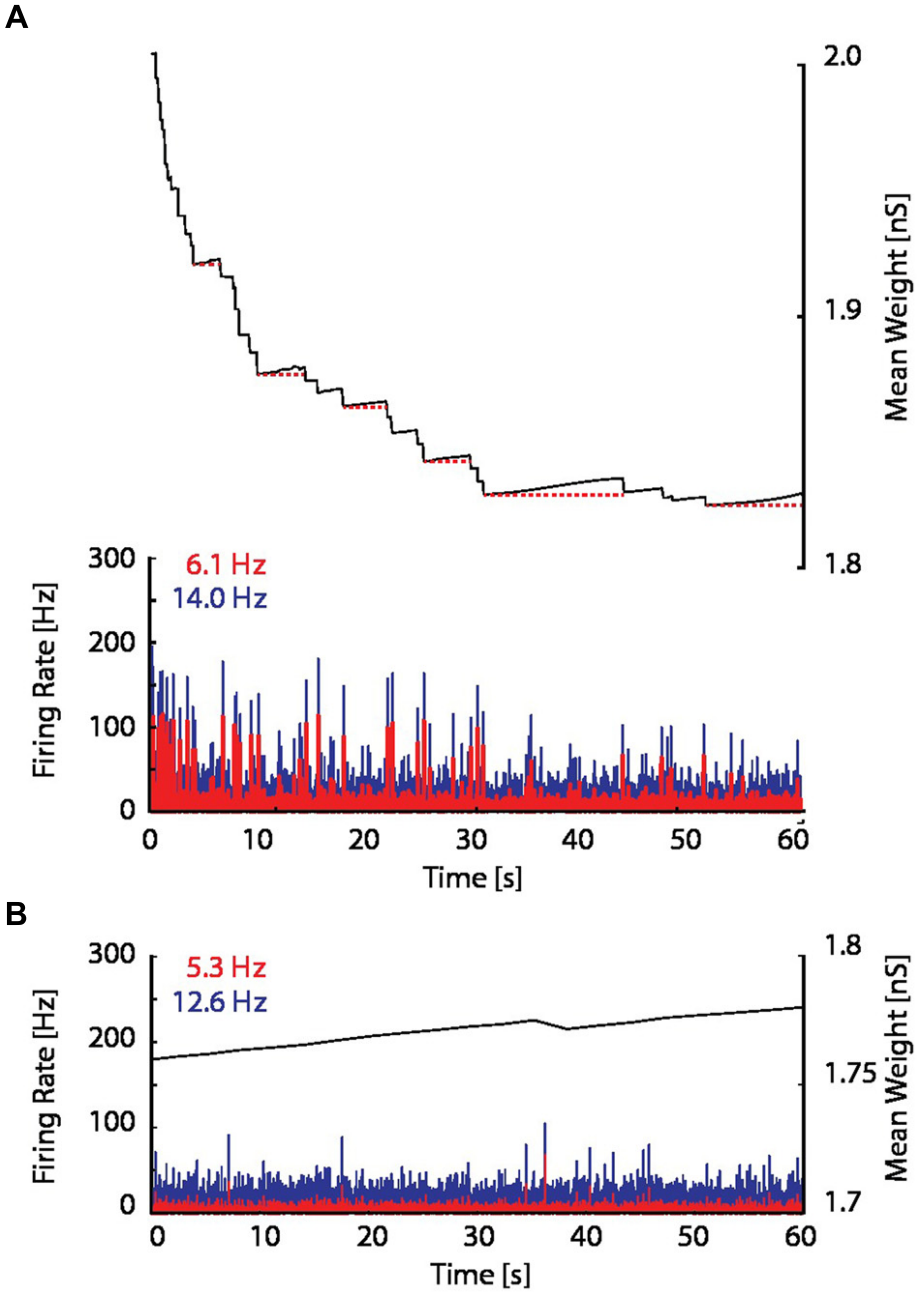
**Excitatory coupling is rising due to on-going synaptic activity. (A)** The mean synaptic weight (black), as in the bottom panel in **Figure 5B**, but with an enlarged scale. Red dotted horizontal lines have been drawn to indicate the progressive increase of the mean synaptic weight between network bursts. **(B)** The firing rate and mean synaptic weight of a network with the resource-dependent STDP rule when the network is initialized in a sub-critical state. The mean synaptic weight is continuously rising, and ultimately will reach the threshold for network bursting.

When the proposed resource-dependent STDP learning rule was introduced into the model, we found that when networks were initialized with strong excitatory–excitatory coupling that caused supra-critical activity and spontaneous network bursting at low rates, the network converged to a critical level (**Figure 5B**) in which regular periodic network bursts were replaced with low frequency irregular bursts. Mean synaptic weights also decreased and stabilized at an intermediate value (**Figure 5B**; bottom panel), consistent with experimental observations (**Figure 6A**; Bear et al., 1987). On the other hand, the mean synaptic weight for sub-critical networks was found to increase toward the same intermediate value (**Figure 6B**). Resource-dependent STDP therefore homeostatically regulates synaptic weights to maintain a mean value just below the threshold for synchronous bursting.

### Self-Organized Criticality Emerges from Resource-Dependent STDP

**FIGURE 7:**
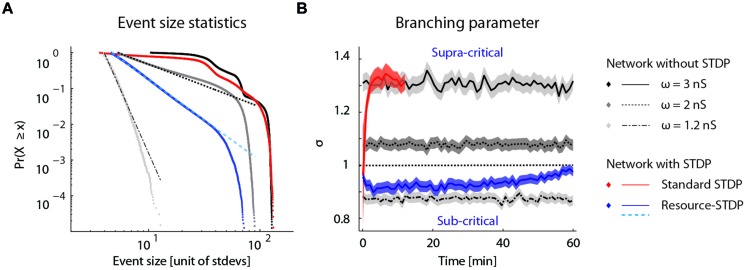
**Self-organized criticality emerges from resource-dependent STDP. (A)** Cumulative probability distribution of network burst magnitudes (Clauset et al., 2009). In networks without STDP, varying the mean synaptic weight of excitatory–excitatory connections (ω = 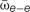) results in different activity regimes: sub-critical with rare aperiodic network bursts (light gray, ω = 1.2 nS), weakly supra-critical with periodic network bursts at rates around 1–10 Hz (dark gray, ω = 2 nS) or strongly supra-critical with periodic network bursts at high rates (black, ω = 3 nS). A standard STDP rule drives the network to a strongly super-critical regime (red), whereas for resource-dependent STDP the resulting burst amplitude statistics follow a power-law (blue). Dashed lines show power-law fits to respective datasets (dot-dashed, ω = 1.2 nS; dashed, ω = 2 nS; cyan-dashed, resource-STDP). **(B)** The branching parameter (σ) is shown for all networks in (a). In networks without STDP, σ does not evolve (gray traces, ω = 1.2, 2, and 3 nS). With a standard STDP rule, σ increases as the network becomes super-critical (red), whereas with the resource-dependent STDP rule a transition from supra-critical to (sub-) critical occurs rapidly and σ further converges toward a value around 1 (blue).

To determine whether the proposed resource-dependent STDP gives rise to what is known for physical systems as a self-organized critical state (Bak et al., 1988), we analyzed the bursting statistics under the different network conditions described above. Self-organized criticality is indicated when the cumulative probability distribution of event amplitudes follows a power law (Beggs and Plenz, 2003). We therefore plotted the cumulative probability of a burst of a given event size occurring for the network dynamics without STDP, with STDP, and with resource-dependent STDP (**Figure 7A**). Without STDP and at the implemented mean excitatory–excitatory synaptic couplings (ω = 1.2, 2, and 3 nS), the burst-size distribution did not follow a power law distribution (**Figure 7A**; gray to black dotted lines). With STDP alone, the distribution was markedly different from a power law (**Figure 7A**; red dotted line). With resource-dependent STDP, all bursts up to 15 times the amplitude of the minimally detected burst followed a power law. The distribution began to deviate when the bursts engaged more than about 20% of the neurons in the network (largest burst involves around 45% of the neurons), equivalent to 14 times the mean network activity (**Figure 7A**; blue dotted line).

Another measure of criticality is the branching parameter (see Materials and Methods), which also gives an indication of the efficiency of the network state to convey information (Beggs and Plenz, 2003; Shew and Plenz, 2013). Networks without STDP have a fixed branching parameter (**Figure 7B**; gray lines). Networks with STDP alone transitioned to a supra-critical state with a branching parameter greater than 1 (**Figure 7B**; red line), and were equivalent to networks without STDP and strong excitatory–excitatory synaptic coupling (**Figure 7B**; solid black line). With resource-dependent STDP, networks converged to a state with a branching parameter around 1, indicating a critical state (**Figure 7B**; blue line).

## Discussion

Spike-Timing-Dependent Plasticity provides a mechanism to modify the synaptic weight of inputs to a neuron according to their relative timing with respect to the back-propagating AP. We report here a phenomenon we refer to as *network-timing-dependent plasticity* (NTDP), whereby local spike-timing-dependent plasticity of individual synaptic pathways is regulated by the relative timing of synchronous bursts generated by the network. NTDP can regulate STDP by blocking (acting in the opposite plasticity direction), saturating (acting in the same direction) and flipping (acting in the same or opposite direction and crossing a threshold of interaction) depending on the relative timing of synchronous network activity. Positive cooperativity (flipping LTD into LTP) could be explained by cooperative interactions between weak and strong inputs (Levy and Steward, 1983; Sjöström et al., 2001), multiple input-driven facilitation of the bAP (Sjöström and Häusser, 2006) or by the *threshold accumulation* of resources (same directions of plasticity), such as intracellular calcium levels (Lisman, 1989; Shouval et al., 2002; Graupner and Brunel, 2010, 2012). In the case of the latter, calcium influxes of individual events would not cross the threshold concentration for LTP and consequently lead to depression, but together more easily cross a threshold concentration for LTP induction (Lisman, 1989). Such a hypothesized positive cooperativity would, however, require a temporal separation to explain why LTD was not flipped into LTP when the burst- and STDP^-^-induced LTD events occurred simultaneously. We further proposed that negative cooperativity (flipping LTP into LTD) could be explained by *threshold depletion* of resources (opposite directions of plasticity) for LTP, such as extracellular calcium levels (Egelman and Montague, 1999). Together, these data suggest a novel mechanism for embedding local timing rules for synaptic plasticity at individual synaptic pathways into global timing rules for synaptic plasticity in the network.

One caveat of the experimental approach here is that severed neuromodulatory axons remaining in the slices could be evoked to release due to MEA stimulation, and could mediate the observed interactions between the two plasticity induction protocols. Recent advances in optogenetic stimulation methods, which can differentially target pyramidal, inhibitory, and neuromodulatory axon populations, could be employed to clarify their role. Also, applying a standard suite of inhibitors for characterizing the signaling pathways involved could further elucidate the biophysical mechanisms at play and could be pursued in follow-up studies as the basis for more detailed biophysical models.

A model in which the negative cooperativity was implemented as resource-dependent STDP was found to homeostatically regulate synaptic weights in an active network, consistent with previous observations of synaptic down-scaling in disinhibited networks (Turrigiano et al., 1998). Stable biological distributions of synaptic weights are the result, even in the presence of synchronous network activity. Moreover, network burst amplitude statistics were power-law distributed reflecting self-organized criticality, a state optimal for information coding (Bak et al., 1988; Beggs and Plenz, 2003; Shew and Plenz, 2013). Self-organized criticality has been observed in various states of vigilance *in vivo* (Petermann et al., 2009; Priesemann et al., 2009, 2013; Hahn et al., 2010), but a plasticity rule to achieve and preserve such a state has thus far been missing. The NTDP rule proposed here offers a candidate solution, and may have implications for the mechanisms underlying pathological network states that occur in epilepsy (Trasande and Ramirez, 2007) as well as the down-scaling of synaptic weights during slow-wave sleep (Massimini and Amzica, 2001; Tononi and Cirelli, 2006; Vyazovskiy et al., 2008).

The present experiments indicate that burst- and STDP^-^-induced LTD share expression mechanisms. The proposed resource-dependent STDP model accounts for LTD at low frequencies of network bursting (Stanton and Sejnowski, 1989; Dudek and Bear, 1992) while preserving the spike-timing dependence of the underlying STDP rule, thus unifying the two phenomena under one mechanism.

Candidate mechanisms for the observed positive cooperativity rely on variables local to the dendrite (Levy and Steward, 1983; Lisman, 1989; Sjöström et al., 2001; Sjöström and Häusser, 2006). An exploration of the impact of combined negative and positive cooperativity, saturation, and blocking effects on the interaction between burst-induced plasticity and STDP would therefore require simulations of detailed neuron morphology and a biophysical STDP rule. Combined with an experimental characterization of the interactions between STDP and LTP induced by high frequency tetanic stimulation (Bliss and Lomo, 1973; Lynch et al., 1983), this approach could reveal a complement to our proposed resource-dependence of STDP, and provide a unifying model for both directionalities of burst induced plasticity, STDP, and their interactions.

## Author Contributions

Experiments were conducted by VD. The resource model was designed and implemented by EM, VD, and MP. Network modeling was performed by EM and MP. System criticality analysis was performed by VD and EM. This manuscript has been written by VD, EM, DK, and HM.

## Conflict of Interest Statement

The authors declare that the research was conducted in the absence of any commercial or financial relationships that could be construed as a potential conflict of interest.
