# Supplementary material for: Network-timing-dependent plasticity
Source: Front Cell Neurosci. 2015 Jun 9;9:220. doi: 10.3389/fncel.2015.00220 (PMC4460533; doi:10.3389/fncel.2015.00220)
Supplement: Supplementary file 1 [file Data_Sheet_1.PDF]

Supplementary Figure 1

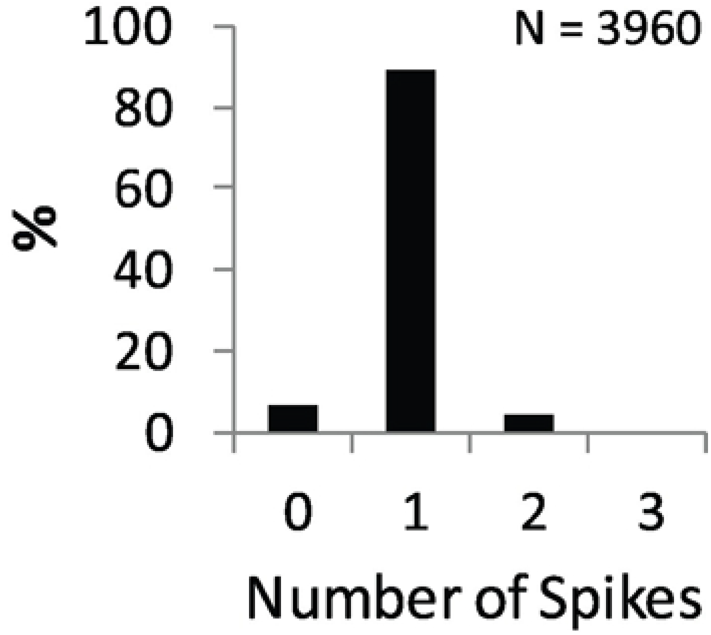

Figure S1 | Probability distribution of the number of evoked spikes due to network bursts. On average, the network burst failed to evoke a spike in 6.8% of the cases, triggers a single spike in 89.2% of the cases, and two spikes in 4% of the cases. We never observed a network burst causing more than two spikes in the patched cells. The latency of the first evoked spike was 3.7 ± 0.2 ms (n = 3960 network bursts recorded in 66 cells).
