# Supplementary material for: Network-timing-dependent plasticity
Source: Front Cell Neurosci. 2015 Jun 9;9:220. doi: 10.3389/fncel.2015.00220 (PMC4460533; doi:10.3389/fncel.2015.00220)
Supplement: Supplementary file 2 [file Data_Sheet_2.PDF]

Supplementary Table 1: Overview of statistical tests for all protocols. Each value is tested against baseline (p-value 1), $STDP^{+}$ protocol (p-value 2), $STDP^{-}$ protocol (p-value 3) and with the 0.1 Hz network bursting protocol (p-value 4). In addition, BSS protocols are compared with their respective burst-STDP protocol (p-value 5).

**Protocol**	# cells	ΔEPSP amp (%)	SEM	Baseline	SDTP+	STDP-	0.1 Hz Bursting	Burst-STDP
				p-value 1	p - value 2	p - value 3	p – value 4	p – value 5
$SDTP^{+}$	11	104	33	**$5.15e^{-3}$**	-	**$3.00e^{-3}$**	**$2.93e^{-3}$**	
$STDP^{-}$	6	-44	11	**$5.16e^{-3}$**	**$3.00e^{-3}$**	-	0.23	
0.1 Hz network bursting	9	-24	20	0.13	**$2.93e^{-3}$**	0.23	-	
**Burst - $STDP^{+}$**								
ΔT = +/- 5 s	8	7	25	$3.94e^{-1}$	**2.20e-2**	0.058	0.16	
ΔT = - 20 ms	7	-30	7	**$2.59e^{-3}$**	**3.01e-3**	0.14	0.04	
ΔT = 0 ms	8	109	65	0.06	0.46	**$3.37e^{-2}$**	**$2.81e^{-2}$**	
ΔT = 50 ms	14	8	21	0.35	**$9.40e^{-3}$**	0.07	0.15	
ΔT = - 20 ms PTX	5	-29	15	0.06	**$1.04e^{-2}$**	0.21	0.43	0.46[1]
**BSS - $STDP^{+}$**								
Δt = - 20 ms	10	62	40	0.078	0.21	**$3.45e^{-2}$**	**$4.19e^{-2}$**	**$4.06e^{-2}$**
Δt = 0 ms	9	46	22	**$3.50e^{-2}$**	0.09	**$3.69e^{-3}$**	**$1.54e^{-2}$**	0.2
Δt = 50 ms	10	31	20	0.078	**$4.15e^{-2}$**	**$7.70e^{-3}$**	**$3.32e^{-2}$**	0.1
**Burst - $STDP^{-}$**								
ΔT = +/- 5 s	7	-34	9	**$4.60e^{-3}$**	**$2.55e^{-3}$**	0.25	0.34	
ΔT = - 50 ms	12	-15	14	0.15	**$1.37e^{-3}$**	0.09	0.34	
ΔT = 0 ms	7	-46	13	**$6.12e^{-3}$**	**$1.64e^{-3}$**	0.46	0.2	
ΔT = 20 ms	9	82	51	0.07	0.36	**3.37e-2**	**$3.38e^{-2}$**	
**BSS - $STDP^{-}$**								
Δt = - 50 ms	7	-12	19	0.271	**$9.68e^{-3}$**	0.09	0.37	0.46
Δt = 0 ms	6	-26	7	**$6.90e^{-3}$**	**$6.45e^{-3}$**	0.09	0.48	0.12
Δt = 20 ms	7	-29	10	**$1.37e^{-2}$**	**$3.37e^{-3}$**	0.16	0.42	**$3.86e^{-2}$**

[1] Burst - $STDP^{+}$ protocol (ΔT = - 20 ms) with intracellular PTX is tested against Burst - $STDP^{+}$ protocol (ΔT = - 20 ms).
